# Nucleosome resection at a double-strand break during Non-Homologous Ends Joining in mammalian cells - implications from repressive chromatin organization and the role of ARTEMIS

**DOI:** 10.1186/1756-0500-4-13

**Published:** 2011-01-21

**Authors:** Preeti Kanikarla-Marie, Sharon Ronald, Arrigo De Benedetti

**Affiliations:** 1 Department of Biochemistry and Molecular Biology and the Feist-Weiller Cancer Center, Louisiana State University Health Sciences Center, Shreveport, LA 71130, USA

## Abstract

**Background:**

The *S. cerevisiae *mating type switch model of double-strand break (DSB) repair, utilizing the HO endonuclease, is one of the best studied systems for both Homologous Recombination Repair (HRR) and direct ends-joining repair (Non-Homologous Ends Joining - NHEJ). We have recently transposed that system to a mammalian cell culture model taking advantage of an adenovirus expressing HO and an integrated genomic target. This made it possible to compare directly the mechanism of repair between yeast and mammalian cells for the same type of induced DSB. Studies of DSB repair have emphasized commonality of features, proteins and machineries between organisms, and differences when conservation is not found. Two proteins that stand out that differ between yeast and mammalian cells are DNA-PK, a protein kinase that is activated by the presence of DSBs, and Artemis, a nuclease whose activity is modulated by DNA-PK and ATM. In this report we describe how these two proteins may be involved in a specific pattern of ends-processing at the DSB, particularly in the context of heterochromatin.

**Findings:**

We previously published that the repair of the HO-induced DSB was generally accurate and occurred by simple rejoining of the cohesive 3'-overhangs generated by HO. During continuous passage of those cells in the absence of puromycin selection, the locus appears to have become more heterochromatic and silenced by displaying several features. 1) The site had become less accessible to cleavage by the HO endonuclease; 2) the expression of the puro mRNA, which confers resistance to puromycin, had become reduced; 3) occupancy of nucleosomes at the site (ChIP for histone H3) was increased, an indicator for more condensed chromatin. After reselection of these cells by addition of puromycin, many of these features were reversed. However, even the reselected cells were not identical in the pattern of cleavage and repair as the cells when originally created. Specifically, the pattern of repair revealed discrete deletions at the DSB that indicated unit losses of nucleosomes (or other protein complexes) before religation, represented by a ladder of PCR products reminiscent of an internucleosomal cleavage that is typically observed during apoptosis. This pattern of cleavage suggested to us that perhaps, Artemis, a protein that is believed to generate the internucleosomal fragments during apoptosis and in DSB repair, was involved in that specific pattern of ends-processing. Preliminary evidence indicates that this may be the case, since knock-down of Artemis with siRNA eliminated the laddering pattern and revealed instead an extensive exonucleolytic processing of the ends before religation.

**Conclusions:**

e have generated a system in mammalian cells where the absence of positive selection resulted in chromatin remodeling at the target locus that recapitulates many of the features of the mating-type switching system in yeast. Specifically, just as for yeast HML and HMR, the locus had become transcriptionally repressed; accessibility to cleavage by the HO endonuclease was reduced; and processing of the ends was drastically changed. The switch was from high-fidelity religation of the cohesive ends, to a pattern of release of internucleosomal fragments, perhaps in search of micro-homology stretches for ligation. This is consistent with reports that the involvement of ATM, DNA-PK and Artemis in DSB repair is largely focused to heterochromatic regions, and not required for the majority of IR-induced DSB repair foci in euchromatin.

## Background

Double Strand Breaks (DSBs) are the most serious form of DNA damage, yet they occur frequently by generation of reactive oxygen species during normal metabolism or by external agents such as ionizing radiation (IR). Furthermore, specific DSBs are genetically programmed, as in immunoglobulin gene rearrangements or during mating type switching in the yeast *S.cerevisiae*. DSBs are generally dealt with by homologous recombination repair (HRR) or non-homologous end-joining (NHEJ). Perhaps the best studied model of DSB repair is the mating type switching of *S. cerevisiae*, which requires the coordinated action of many proteins, and minimally consists of the HO endonuclease and a host of components required for a gene conversion event that utilizes the silent mating cassette of the opposite type as the donor (for a review, see [1]). Following specific cleavage at the MAT locus by the HO endonuclease, the recessed strand is further processed by an endonuclease and/or exonuclease to generate long stretches of ssDNA capable of strand invasion and then as acceptors for HR replication/repair utilizing the information from HML or HMR as the template. The precise involvement of the nuclease in the resection process is unclear, but Sae2 has been implicated [2]. However, clearly the process is complex and highly regulated since, for example, strand resection is suppressed in a strain deleted for the HML and HMR [1]. In that case, repair occurs only via NHEJ and with minimal processing of the ends, which are then capped by yKu and the DSB-binding complex Mre11/Rad50/Xrs2p. The silent cassettes (HML and HMR) are characteristic heterochromatic regions that are established by several factors, and minimally by the four Sir proteins, Rap1 and ORC, and resulting in repressive chromatin. This includes suppression of transcription of genes present at or near HML and HMR, and inaccessibility to cleavage by the endogenous HO nuclease at HML and HMR. In fact, the activity of HO is strongly influenced by the chromatin state of its target sequence.

In mammalian cells, NHEJ is the predominant pathway of DSB repair, and although NHEJ-defective cell lines show marked defects in DSB repair and sensitivity to IR, cells defective in ATM, a key protein kinase involved in repair and cell cycle arrest, repair most DSBs normally [3]. Thus, NHEJ can function in the absence of ATM signaling. However, 10-20% of visible IR-induced DSBs (repair foci) are repaired with slow kinetics and appear to require ATM and a target nuclease, Artemis, involved in ends processing [4]. Interestingly, these DSBs that persist in the presence of an ATM inhibitor localize to heterochromatic regions associated with markers of silenced chromatin, suggesting that ATM and Artemis are particularly important for repair of DSBs at condensed genomic regions [3]. Artemis is an ATM-dependent substrate after radiation [3]. Artemis is both an endouclease and exonuclease that has the capacity to cleave hairpin-ended DSBs and to remove 3'- or 5'-single-stranded overhangs, following binding of the DNA ends by the DNA-dependent protein kinase (DNA-PK) [4]. Furthermore, at least *in vitro*, the endouclease and exonuclease trimming are inversely regulated by DNA-PK, and depending on the type of ends (e.g., incompatible or blocked) [5]. Hence, the activity of PI3K-like kinases (PIKKs) can significantly affect the processing of DSB ends by Artemis, and particularly in the context of heterochromatic regions. Recent work has also indicated that Artemis is directly involved in generating the classic DNA fragmentation ladder obtained during apoptosis [6]. One hallmark of apoptosis is DNA degradation that first appears as high molecular weight fragments, followed by extensive internucleosomal fragmentation. DNA-PK is typically involved in the repair of DSBs by facilitating processing of damaged ends, but in the context of apoptotic conditions, it seems that DNA-PK and Artemis' roles shifts to that of final executioners [6].

We have recently described a system of DSB repair where we have transposed the minimal yeast HO endonuclease system to mammalian cells, by generating cells containing the HO target sequence and utilizing an HO-encoding adenovirus [7,8]. In this system, cleavage was very efficient (depending on the MOI), and repair occurred via simple ends-joining during a time course of infection - the HO enzyme is largely degraded at later times, thus avoiding continuous recutting of the site. While the repaired junctions were not isolated and re-analyzed from individual clones, the repair appeared to be predominantly a high fidelity religation of the 4-bp cohesive ends at the level of PCR-generated band. The HO target construct is integrated, and hence, we had not maintained the puromycin selection for many passages, as this did not appear necessary. We now report some interesting changes that occurred in these "unselected" cells that can be best explained by the establishment of heterochromatic, repressive chromatin at the locus.

## Results and Discussion

**Figure 1:**
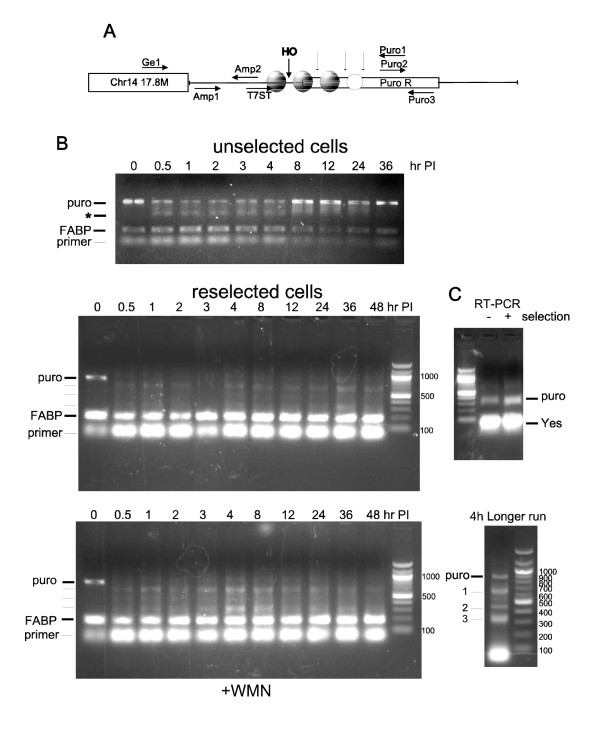
**Pattern of cleavage and repair generated with Adeno-HO nuclease**. A) Diagram of the integrated HO target cassette, including a representation of the HO cleavage site, position of the primers used, and putative positioning of nucleosomes and secondary cleavage sites. B) Kinetics of cleavage and repair (hours post-infection [PI] with adeno-HO) in unselected and puromycin reslected cells. For these experiments, 10,000 cells were infected at an MOI of 200 in 96-well plates, and the DNA was isolated at the indicated times with Wizard genomic DNA isolation kit (Pormega). The product of the original target (amplicon T7ST/Puro 1) is indicated for cells at the start of infection as "puro". Other intermediate PCR bands are indicated by lines. FABP is a PCR product from a single-copy gene on mouse chromosome 6. Wortmannin (WMN; 30 μM) was added to the cells in the bottom panel throughout the time course post-infection. The products from 4h time point of the bottom panel were separated on a new gel to the right, using more material and a longer gel run. The two PCR reactions (Puro and FABP) were run in parallel with the same thermocycle settings and the products were mixed before loading each lane. C) RT-PCR for semi-quantitative analysis of the expression of puro mRNA. First-strand cDNA was produced with oligo-dT from 5 μg of total RNA from unselected and reselected cells. Puro mRNA and Yes mRNA were amplified in parallel for estimation of their relative expression.

We recently resurrected the Adeno-HO/MM3MG system to address one simple question: What would be the effect of adding wortmannin (WMN), a general inhibitor of PIKKs including ATM, ATR, and DNA-PK on the kinetics of repair of the single HO-mediated DSB? A diagram of the HO target cassette is shown in Figure 1A. The kinetics of cleavage and repair can simply be monitored as a loss and then recovery of the T7ST/Puro1 amplicon generated with primers flanking the HO cleavage site. Much to our dismay, very little cleavage was obtained at 200 MOI (Figure 1B), which was different than our previously published work [7,9]. In that work, cleavage was complete within 0.5-1 h post-infection (PI), and repair began variably after 3-10 h. We knew from other work ongoing in the lab that the virus stock was active, so that could not be a reason for the loss of cutting. Some cleavage was evident, but this did not appear to involve more than 50% of the cells. Where cleavage was detected, repair was complete by 8h PI. There appeared to be also a confusing smaller band of about 500 bp (asterisk), which we could not interpret at the time. This band was present only after inducing the DSB and it seemed to be a specific repair product. This product was less obvious at 36h PI, which corresponds to about 1.5-2 cell divisions later.

**Figure 2:**
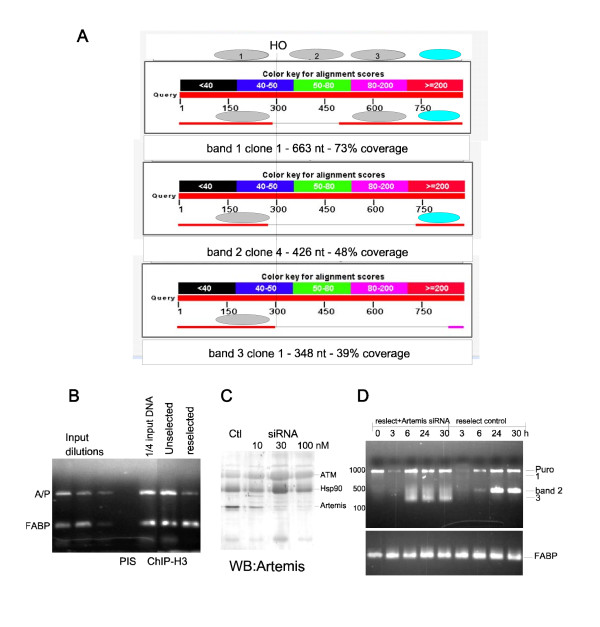
**Extent of nucleosomes loading at the HO-puro locus in unselected and reselected cells, and the role of Artemis**. A) Graphical sequence alignment of representative clones along the T7ST-Puro1 sequence - obtained with "Align 2" of NCBI BLAST. B) Histone H3 occupancy in unselected and reselected cell by ChiP. Chromatin was formaldheide cross-linked from 10^6 ^cells and ChIP for H3 was carried out as described in [7]. Input dilutions are from DNA isolated from 5000 cells and 3-fold dilutions thereof. PIS is the CHIP protocol carried out with pre-immune serum. ¼ input DNA is product generated by cross-link reversal from ¼ the material used for ChIP. Amp/Puro is the band generated with primers Amp1 and Puro1. C) Western blot for Artemis was carried out from reselected cells, before and after 24h treatment with siRNA against Artemis (purchased from Dharmacon). The blot was sequentially probed for Hsp90 and ATM. All antisera were from Abcam. D) Kinetics of cleavage and repair (HO time course of infection) with reselected cells treated or not with Artemis siRNA.

Since the HO nuclease is very sensitive to chromatin structure, we suspected that perhaps the site had become heterochromatic and hence partially inaccessible to cleavage. We then reapplied selection with puromycin (1 μg/ml). Nearly 80% of cells died, but this obviously did not involve general ejection of the construct, as this was clearly present by PCR. It seemed more likely that the locus had become transcriptionally inactive in the absence of selective pressure. The remaining 20% began dividing rapidly after a short lag and recovered fully in a few days. They were maintained under selection and named "reselected cells" (Figure 1B, C). We then repeated the infection/cleavage experiment with these cells, and also tested for the expression of the puro mRNA. Total RNA from unselected and reselected cells was isolated and first strand cDNA was obtained using olgo-dT, followed by semi-quantitative PCR with specific primers. As we had suspected, the expression of puro mRNA was significantly lower in the unselected cells in comparison to the reselected (Figure 1C) - the expression of Yes mRNA was used as a normalization control. The simplest explanation for these results is that the locus had become heterochromatic and partially silenced in the absence of selection, in at least a large fraction of the cells. We then carried out a time course of cleavage and repair in the reselected cells. This time cleavage was very efficient and essentially complete within ½ hr. Repair also began almost right away but did not reach the maximum until 4-8h later. The greater accessibility to HO nuclease could thus explain the more complete cleavage at the HO site, consistent with a possible decondensation of chromatin at the locus and increased puro expression following reselection. However, there was a second surprise. Apparently, only a fraction of cells (less than 50%) repaired the DSB with regeneration of the correct sized PCR product (labeled "puro" in Figure 1B). The remainder generated a short ladder of three smaller and discrete PCR products (Figure 1B - 4h longer run). Curiously, the spacing between these bands was highly reminiscent of a nucleosomal ladder with a separation of ~200 bp. However, the spacing between bands 2 and 3 is only 80-120 bp, suggesting that this segment might be occupied by a non-standard nucleosome or some other type of protein or protein complex. We will refer to that as "nucleoprotein complex 4". These PCR products were better separated on another gel in the right panel, and bands 1, 2 and 3 were excised and re-amplified. Upon close inspection, the PCR bands were not completely sharp, so they were shot-gun cloned in the *Sma*I site of Bluescript plasmid. Several clones obtained from each band were sequenced. Clones derived from each PCR band showed very high sequence identity of >98% along their coverage, with the main differences lying within the gaps at the breakpoints. The alignment of one representative clone from each band with the original HO-puro sequence is shown in Figure 2A. The gaps suggested spacing for the nucleosomes in the region, where the sequence flanking the T7ST primer and including the first nucleosome, to the left of the HO, was preserved. In contrast, progressively larger gaps on the right side of the HO site suggested removal of the next two nucleosomes and the following nucleoprotein complex 4. It is possible that the internucleosomal resection occurred beyond the boundaries of the T7ST and Puro1 amplicon in some cells; only such events would not have been picked up without using more distal primers. In summary, the specific pattern of cleavage and repair suggested that, unlike the precise regeneration of the segment at the HO joint obtained when the cells were first made, the reselected cells often repaired the DSB only after further resection, which apparently involved the removal of full nucleosomal segments. We should however caution that other explanations for this pattern of cleavage and religation are possible beside removal of nucleosomes. For example, there could be a specific pattern of DNA bending in that region that renders it locally exposed to discrete nucleolytic cuts. One distinct possibility is that the cuts do not occur within the linker segments, as we have drawn in Figure 2A, but rather are rotationally phased on the surface of the nucleosomes. In such case, the nucleosomes would still have to be spatially organized in the cell population but for instance, they may not be positioned where we drew them. In this respect, we should also stress that we do not claim that the ~500 bp PCR product obtained in Figure 1B with the unselected cells (top panel) is the same as bands 1 or 2 of the reselected cells (bottom panels). While this is clearly possible, in reality the organization of nucleosomes in the two cell populations may be different, and hence produce bands that are only similar in size. A last caveat that we must suggest is that the PCR products may be to some extent an artifact. While it seems clear that they do represent secondary cleavage events after the main HO cut, it is possible that the PCR conditions would favor the amplification of only a few products, and that in reality the nucleolytic processing in the population is more heterogeneous. Multiplex PCR is very efficient, and up to 5 products have been independently and reliably amplified in published research. However, only one primer pair was used here, and hence the possibility of preferential amplification remains.

The addition of WMN during the time course of infection did not alter much the kinetics of cutting and repair, but the smaller bands appeared more defined and somewhat less smeary. We discuss later how this may be the effect of a shift in the activity of Artemis toward greater endonucleolytic performance and less exonucleolytic.

Evidence for formation of repressive chromatin was obtained by showing that expression of the puro mRNA was reduced in unselected cells, and then restored to higher level after reselection. To seek direct evidence for a more condensed chromatin in the unselected cells compared to reselected, we determined by ChIP the histone H3 occupancy at the HO cassette. To obtain a larger snap-shot of the region and maximize the difference in nucleosomal load between unselected and reselected cells, primers Amp1 and Puro1 were used, which encompass a ~2 kb segment. The results of a representative experiment are shown in Figure 2B, which revealed a ~3-fold stronger ChIP signal from unselected vs. reselected cells. The data were compared to the signal obtained from the FABP amplicon, which resides in a gene that is not transcriptionally active in these cells. These data further support the idea that the region spanning the engineered HO-puro cassette had become heterochromatic and silenced, hence more stably loaded with nucleosomes, in the absence of selective pressure. Additional evidence for this could come from determination of repressive chromatin marks (e.g., histone H3K9me3 or DNA methylation) in future experiments.

The involvement of Artmeis in generating the repair ladder was hypothetical, somewhat corroborated by the enhancing effect of WMN in this experiment. Clearly, there was some downstream effect from inhibition of one or more PIKK, and Artemis is known to be regulated by both ATM and DNA-PK. It was also reported that *in vitro *the endonucleolytic and exonucleolytic activities of Artemis are oppositely regulated by DNA-PK [5]. We thus decided to test if Artemis, which is not an essential protein, was in fact involved in this process. Knockdown of Artemis was induced with siRNA, and 80% reduction was obtained with 100 nM of oligo (Figure 2C). The same blot was also probed for ATM to verify presence of this critical protein in the process, and Hsp90 as loading control. We then pre-treated the cells for one day with siRNA and repeated the infection with Adeno-HO with selected time points. Without the siRNA, the experiment gave essentially the same results as in Figure 1B, with again a prominent band of ≤500 bp. There was a hint of bands 1 and 3, more noticeable at the 3h PI early time point, but since they are less intense than those in Figure 1B, it suggests that ends-processing may be a stochastic process, possibly dictated by the predominant chromatin organization in the population at the time of the experiment. The two experiments were carried out within two weeks of each other - 4 passages. In cells knocked down for Artmeis, there was instead an indistinct smear of products between ~600 and 200 bp. Hence, while clearly there was nucleolytic activity near the DSB, there was no evidence for generation of a distinct laddering pattern. This experiment suggested that Artemis may be involved in a specific pattern of ends-processing at the DSB, consisting of internucleosomal, endonucleolytic release of fragments adjacent to the HO cut site before religation. This pattern was somewhat enhanced by inhibition PIKKs, which presumably alters the specificity of Artemis. In the absence of Artemis (knock-down experiment) the ends are apparently processed more heterogeneously, likely by a combination of other endo- and exo-nucleases. For instance, the Mre11 nuclease component of the DSB-binding complex MRN could provide such function [10]. While this paper was undergoing review, Helmink et al. reported that presence of γH2AX in chromatin flanking the DSBs generated by the RAG recombinase during V(D)J recombination affects ends resection and processing by Artemis and CtIP [11].

## Conclusions

In this paper we report a few new findings using a system adapted from yeast to introduce a single genomic DSB in mammalian cells. First, as in yeast, the cleavage at the genomic site appears influenced by the organization of chromatin. It is more accessible when the site is euchromatic, and less accessible when the locus becomes more heterochromatic and transcriptionally silent. Second, in the reselected cells, we identified a novel pattern of processing and repair at the junctions, which generated a curious and prominent PCR ladder, which we interpret as removal of DNA segments bound to nucleosomes or other protein complexes. If we are right, first, the nucleosomes must be positioned in the majority of the cells for this to occur. Second, this is clearly different from the pattern of accurate repair originally seen when the first passages of MM3MG-HO cells were analyzed. In those cells, there was one prominent PCR product identical in size and sequence to that of uncut cells. It is tempting to speculate that in the original cells, the locus was even less packed with nucleosome, although histones were clearly present [7]. Alternatively, the nucleosomes were more readily evicted during DSB repair [7]. We hypothesize instead, that in the reselected cells, the repair now requires more extensive remodeling of the locus and the resection of adjacent nucleosomes, and that this happened after the establishment of repressive chromatin in the absence of selection. The involvement of Artemis in the internucleosomal endonucleolytic cleavage at either side of the DSB is also a novel issue, *albeit *incompletely resolved. Artemis was previously reported to be partly responsible for generating the apoptotic ladder [6], and in fact, cells lacking Artemis lack or show delayed kinetics of appearance of the typical banding induced by apoptosis [6]. We suggest that Artemis is responsible for producing the ladder we observe in our reselected cells, and that this activity might be important to create short segments of DNA ends that are better suitable for religation. The role of chromatin at DSBs is clearly a known example of how Artemis may be particularly involved in processing of perhaps only a fraction of IR-induced DSBs, which are however localized to heterochromatin [3,12]. We should point out, however, that Goodarzi et al. suggested that Artemis' action is particularly involved in HRR during the G2 phase of cell cycle [12]. In contrast, our work suggests that Artemis is clearly involved in NHEJ and in unsynchronized cells (hence largely in G1-S). It was possibly a lucky accident that the original clone of puromycin-selected cells with that specific HO integration site, which we ended up selecting for our studies, displayed plasticity in chromatin organization at the locus.

## Competing interests

The authors declare that they have no competing interests.

## Authors' contributions

PKM and ADB contributed Figure 1. SR and ADB contributed Figure 2. ADB wrote the paper with the help of PKM and SR. All authors have read and approved the manuscript.
